# Kinetics of release of antiretroviral drugs through a silicone intravaginal ring

**DOI:** 10.1186/1471-2334-14-S2-P1

**Published:** 2014-05-23

**Authors:** Bruno Pozzetto, Meriam Memmi, Marlène Desloir, Blaise Figuereo, Thomas Bourlet, Christian Carrot

**Affiliations:** 1 University Hospital of Saint-Etienne, Groupe Immunity of the Mucous Membranous and Pathogen Group (GIMAP), EA 3064, Saint-Etienne, France; 2 University Hospital of Saint-Etienne, Engineering of polymer materials, Saint-Etienne, France

## Aim

The propagation of HIV-1 infection represents a major problem of public health, notably in sub-Saharan Africa where about six out of ten infected adults are women. Among the new options of preventive therapy, antiretroviral drugs used as microbicides distributed via an intravaginal ring (IVR) constitutes an original solution. IVR is an interesting mode of drug delivery because it is a coit-independent device that can be left in the vagina for several months and can release regular amounts of microbicides. The aim of this work was to set up a silicone IVR able to deliver simultaneously several drugs with the precise objective of determining if the release of each drug is possible with the used silicones and of choosing the best formulation.

## Materials and methods

IVRs were made manually with siloxanes combining two elastomers from Blue Star Silicone before and after reticulation, and were loaded with different concentrations of tenofovir. The rings were then placed under conditions mimicking closely the physiological environment of the vagina. The amounts of released tenofovir were measured by UV spectrophotometry.

## Results

The manual mode of fabrication of IVR is described with details. Different combinations of siloxanes were used. Only some of them were shown to release amounts of tenofovir that can be considered as efficient for protecting from a heterosexual HIV-1 infection. We present rheological analyses that sustain these differences.

## Conclusions

We propose a model that was shown able to test the release of antiretroviral drugs by a silicone multi-reservoir IVR under various experimental conditions. The release of tenofovir by silicone obeys to physico-chemical rules that we tried to elucidate. The study of other antiretroviral drugs is ongoing with the aim of developing a device able to protect efficiently against the viral loads observed in semen. At term, this system could be rendered more complex by the addition of antiviral compounds directed against other viruses and/or of anti-inflammatory drugs or hormones.

